# Mechanisms of Cisplatin in Combination with Repurposed Drugs against Human Endometrial Carcinoma Cells

**DOI:** 10.3390/life11020160

**Published:** 2021-02-19

**Authors:** Chi-Kang Lin, Shu-Ting Liu, Zih-Syuan Wu, Yu-Chi Wang, Shih-Ming Huang

**Affiliations:** 1 Department of Obstetrics and Gynecology, Tri-Service General Hospital, National Defense Medical Center, Taipei City 114, Taiwan; kung568@mail.ndmctsgh.edu.tw; 2 Department of Biochemistry, National Defense Medical Center, Taipei City 114, Taiwan; shuting0719@gmail.com (S.-T.L.); g8401011@gapps.ndmctsgh.edu.tw (Z.-S.W.)

**Keywords:** cisplatin, reactive oxygen species, repurposing drugs, cytotoxicity, p53

## Abstract

Although endometrial carcinoma is one of the most common gynecological malignancies worldwide, its precise etiology remains unknown. Moreover, no novel adjuvant and/or targeted therapies are currently being developed to achieve greater efficacy for endometrial cancer patients who develop chemotherapeutic drug resistance. In this study, we used three human endometrial cancer cell lines, RL95-2, HEC-1-A, and KLE, to investigate the responsiveness of cisplatin alone and in combination with potential repurposed drugs. We first found that RL95-2 cells were more sensitive to cisplatin than HEC-1-A or KLE cells. The cytotoxicity of cisplatin in RL95-2 cells may reflect its ability to perturb the cell cycle, reactive oxygen species production and autophagy as well as to induce senescence and DNA damage. Similar effects, although not DNA damage, were also observed in HEC-1-A and KLE cells. In addition, downregulation of p53 and/or cyclin D1 may also impact the responsiveness of HEC-1-A and KLE cells to cisplatin. We also observed that resveratrol, trichostatin A (TSA), caffeine, or digoxin increased the apoptotic process of cisplatin toward RL95-2 cells, while amiodarone or TSA increased its apoptotic process toward HEC-1-A cells. The combination index supported the assertion that the combination of cisplatin with caffeine, amiodarone, resveratrol, metformin, digoxin, or TSA increases the cytotoxicity of cisplatin in HEC-1-A cells. These findings suggest potential strategies for enhancing the efficacy of cisplatin to overcome drug resistance in endometrial carcinoma patients.

## 1. Introduction

Endometrial cancer is the most common of the gynecologic malignancies, with a worldwide prevalence rate estimated to be 10–20% [1]. The precise etiology of endometrial cancer is unknown, and most cases are sporadic. The lesions are usually confined to the uterus, and standard treatment consists of primary hysterectomy and bilateral salpingo-oophorectomy, as well as adjuvant radiotherapy and chemotherapy in patients with risk factors [2]. The surgical approaches are mostly minimally invasive laparoscopic or robotic techniques or laparotomy. In patients without metastasis, the 5-year overall survival rate ranges from 74% to 91% [3]. However, although the overall survival rate is relatively good for patients diagnosed early, the recurrence rate is reportedly 7.7% to 63.3%, and is associated with surgical-pathological risk factors [4]. Up to now, no novel adjuvant and/or targeted therapies have been developed to achieve greater treatment specificity for these patients or to overcome chemotherapeutic drug resistance.

To develop more individualized treatments, there is good evidence for the use of surgical pathological staging and histologic subtyping to classify endometrial cancer patients as high- or low-risk. The most common tumors (type I) are usually low grade, endometrioid type, and estrogen-dependent, and often have an excellent prognosis. By contrast, type II tumors, serous, undifferentiated, and clear cells, are generally high grade with a tendency to recur, even when treated early [5]. A broad array of molecular alterations has been identified in endometrial cancers, among which several affecting oncogenes, tumor suppressor genes, transcription factors, and DNA repair genes appear to have a prognostic value and to provide the potential for development of targeted therapies [6]. Type I tumors also show microsatellite instability and PTEN (Phosphatase and Tensin Homology) mutations. PTEN is a tumor suppressor gene that negatively regulates the PI3K/AKT (Phosphatidylinosito 3-kinase/a serine/threonine protein kinase) signaling pathway, mutations within which contribute to the pathogenesis of endometrial carcinoma. Type II tumors exhibit p53 mutations and chromosomal instability [7]. p53 mutations are predictive of prognosis in endometrial cancer and are associated with unfavorable outcomes [8]. Considering all the various genes and signaling pathways potentially involved in the pathogenesis of endometrial cancer, strategies for targeting them would seem to be a reasonable therapeutic approach.

The primary cause of death from endometrial cancer is disease recurrence, leading to progressive growth of the tumor [9]. Although chemotherapy or radiotherapy can target most tumor cells, relapse occurs in many cases because of drug resistance. Cisplatin is a well-known chemotherapeutic drug that acts by crosslinking with the N^7^ of guanine in DNA [10]. Its mode of action is related to its ability to interfere with DNA repair mechanisms, cause DNA damage, and then induce apoptosis in cancer cells [11]. In recent years, much research has focused on the role of oxidative stress, defined as an imbalance between reactive oxygen species (ROS) and antioxidants, in the pathophysiology of malignant transformation in endometriosis [12,13,14,15]. But oxidant/antioxidant imbalance is a double-edged sword that can promote both carcinogenesis and cancer cell death. Cisplatin induces ROS that triggers cell death [16]. On the other hand, cancer cells frequently develop resistance to cisplatin that has been attributed to three molecular mechanisms: increased DNA repair, altered cellular accumulation, and increased drug inactivation [17]. One approach to overcoming cisplatin resistance, as well as its toxic side effects, has been the use of other platinum-containing anti-cancer drugs, such as carboplatin and oxaliplatin, but the benefit over cisplatin has been limited [18]. A strategy of drug repurposing could potentially overcome the drug resistance to cisplatin and reduce its toxicity during cancer therapy.

A significant challenge to drug repurposing is choosing the optimal therapeutic target. This can be achieved in part by using cancer cell lines to increase our understanding of the biological mechanisms of carcinogenesis. Here, we used three endometrial cell lines, RL95-2 (type I), HEC-1-A (type II), and KLE (type II) to address the selectivity of cisplatin chemotherapy, its working mechanisms, and its potential combination with repurposed drugs related to p53-dependent pathways, including caffeine, digoxin, trichostatin A (TSA), amiodarone, resveratrol, and metformin previously used in our laboratory [19,20,21,22,23]. We hope that this study might provide novel insights into the functional mechanisms of cisplatin and drug combinations that could be potentially applied to clinical endometrial carcinoma therapy.

## 2. Methods and Materials

### 2.1. Cell Culture and Reagents

RL95-2 (ATCC^®^CRL-1671^™^), HEC-1-A (ATCC^®^HTB-112^™^), and KLE (ATCC^®^CRL-1622^™^) human endometrial carcinoma cell lines were purchased from the American Type Culture Collection (ATCC; Manassas, VA, USA). RL95-2 and KLE cells were cultivated in Dulbecco’s Modified Eagle’s Medium Nutrient Mixture F-12 (DMEM/F12). HEC-1-A cells were cultivated in McCoy’s 5A medium supplemented with 10% fetal bovine serum (FBS) and 1% penicillin-streptomycin (Thermo Fisher Scientific, Waltham, MA, USA). Amiodarone, caffeine, cisplatin, 2′,7-dichlorofluorescein diacetate (DCFH-DA), digoxin, metformin, oxaliplatin, propidium iodide (PI), resveratrol, thiazolyl blue tetrazlium bromide (MTT), and trichostatin A (TSA) were obtained from Sigma Aldrich (St. Louis, MO, USA).

### 2.2. Cell Survival Analysis

Cells were seeded into 24-well culture plates and incubated for 1 day, after which they were exposed to the indicated drugs in fresh DMEM for the indicated periods of time. After adding MTT solution (0.5 mg/mL in phosphate buffered saline (PBS)) to each well, the cells were incubated for 1 h at 37 °C. Dimethyl sulfoxide (DMSO; 200 μL) was then added, and the absorbance at 570 nm and 650 nm were measured using an enzyme-linked immunosorbent assay (ELISA) plate reader (Multiskan EX, Thermo Fisher Scientific, Waltham, MA, USA). A control group containing cells cultured in medium only was defined as 100% metabolic activity. Combination index (CI) was calculated using CalcuSyn (Biosoft, Cambridge, UK) to generate the isobologram as previously described [24]. In general, CI < 1 indicates a synergistic combination effect and CI > 1 indicates an additive combination effect [25].

### 2.3. Fluorescence-Activated Cell Sorting (FACS), Cell-Cycle Profiles, ROS, and Senescence Analyses

Cell cycle profiles were evaluated based on cellular DNA content using FACS. Cells were fixed in 70% ice-cold ethanol and stored at −30 °C overnight, after which they were washed twice with ice-cold PBS supplemented with 1% FBS and stained with PI solution (5 μg/mL PI in PBS, 0.5% Triton x-100, and 0.5 μg/mL RNase A) for 30 min at 37 °C in the dark.

The fluorescent marker DCFH-DA was used to determine intracellular ROS levels. Cells were incubated for 24 h with different concentrations of cisplatin or oxaliplatin. Living cells were then stained with DCFH-DA (10 μM) for 40 min at 37 °C and harvested. After washing the cells once with PBS, they were evaluated using a FACSCalibur flow cytometer and Cell Quest Pro software (BD Biosciences, Franklin Lakes, NJ, USA).

For flow cytometric senescence assays, senescence associated β-Gal activity was measured using the fluorescent substrate, 5-dodecanoylaminofluorescein di-β-d-galactopyranoside (C_12_FDG), (Invitrogen, Carlsbad, CA, USA) according to the manufacturer’s instructions. Briefly, the cells were seeded into 6-well culture plates and treated for 24 h with various concentrations of cisplatin. After the incubation, the cells were harvested, washed twice with PBS, and stained with 33 μM C_12_FDG for 15–20 min at room temperature. Fluorescence intensity was then evaluated using a FACSCalibur flow cytometer and Cell Quest Pro software (BD Biosciences, Franklin Lakes, NJ, USA).

### 2.4. Western Blotting

RL95-2, HEC-1-A, or KLE cells were lysed in Radio-immunoprecipitation Assay buffer (100 mM Tris-HCl (pH 8.0), 150 mM NaCl, 0.1% SDS, and 1% Triton 100) at 4 °C. Proteins in the resultant lysates were separated by sodium dodecyl sulfate polyacrylamide gel electrophoresis (SDS-PAGE) and analyzed by immunoblotting with antibodies against α-actinin (ACTN), β-actin, DRP1, FAS, MFN1, Nrf2, p53, p62, PCNA, PGC-1α, TFAM, TOM20 (Santa Cruz Biotechnology, Santa Cruz, CA, USA), p-DRP1, LC3B, cleaved poly-ADP-ribose polymerase (PARP) (Cell Signaling, Danvers, MA, USA), Cyclin D1, γH2A.x (Abcam, Cambridge, UK), and HO-1 (Enzo Life Sciences, Farmingdale, NY, USA).

### 2.5. Evaluation of Mitochondrial Morphology

RL95-2 and HEC-1-A cells were seeded into 12-well plates at a density of 10^5^ cell/well. After treatment with the indicated concentrations of cisplatin for 24 h, the cells were washed, incubated with 10 nM MitoView^TM^ Green (Biotium, CA, USA) at 37 °C for 15 min, and washed again three times with PBS. Mitochondrial morphology in each group was observed using LeadView 2800AC-FL microscope (Leader Scientific Co. Ltd., Taiwan, China) equipped with a 400× objective and analysed using Image-Pro^®^Plus (Media-cybernetics, USA) [26]. Green fluorescence reveals the mitochondria stained by MitoView^TM^ Green.

### 2.6. Statistical Analysis

Values are expressed as the mean ± SD of at least three independent experiments. All comparisons between groups were made using Student’s *t*-tests. Statistical significance was set at *p* < 0.05.

## 3. Results

### 3.1. Differential Responsiveness to Cisplatin and Cell-Cycle Profile of Three Endometrial Cancer Cell Lines

**Figure 1:**
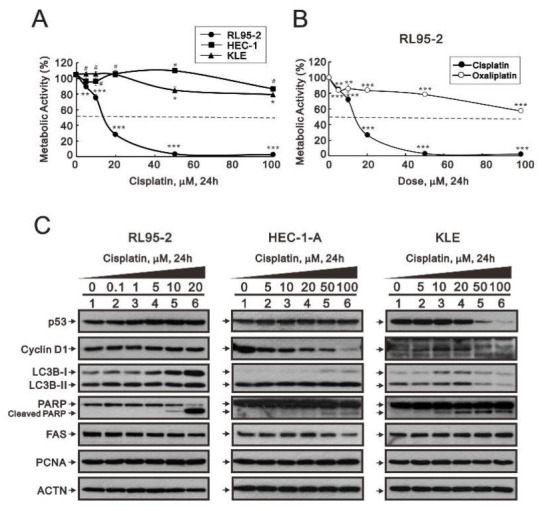
Responsiveness of human endometrial carcinoma cells to cisplatin. (**A**–**C**) Three human endometrial carcinoma cell lines, RL95-2, HEC-1-A, and KLE, were incubated for 24 h with the indicated concentrations of cisplatin or oxaliplatin. (**A**,**B**) Metabolic activity measured using the MTT (thiazolyl blue tetrazlium bromide) method for cisplatin (**A**) in RL95-2, HEC-1-A, and KLE cells and for oxaliplatin (**B**) in RL95-2 cells. Trend of three independent experiments was shown. (**C**) Cell lysates were subjected to western blot analysis using antibodies against the indicated proteins. Alpha actinin (ACTN) was the protein loading control. The results are representative of three independent experiments. ^#^ *p* > 0.05, * *p* < 0.05, ** *p* < 0.01, and *** *p* < 0.001 (Student’s *t*-tests).

To better understand the differential responsiveness of endometrial cancer to cisplatin and the potential combination therapy, we assessed its effects on three commercial endometrial cancer cell lines, RL95-2 (type I), HEC-1-A (type II), and KLE (type II). RL95-2 cells are epithelial-like and/or rounded carcinoma; HEC-1-A and KLE cells are epithelial adenocarcinoma. Using MTT (thiazolyl blue tetrazlium bromide) cell survival assays, we observed that RL95-2 cells were more sensitive to cisplatin than HEC-1-A or KLE cells (Figure 1A). By contrast, RL95-2 cells were much less sensitive to oxaliplatin (Figure 1B) and the responsive pattern of oxaliplatin was similar to the pattern of cisplatin in HEC-1-A or KLE cells (supplementary Figure S1). In addition, using Western blotting we examined the cisplatin-induced changes in endogenous levels of p53 (a biomarker for cell cycle and apoptosis), cyclin D1 (a biomarker for cell cycle), LC3B (a biomarker for autophagy), PARP (poly-ADP-ribose polymerase) (a biomarker for apoptosis), FAS (fatty acid synthase) (a biomarker for fatty acid synthesis), and PCNA (proliferating cell nuclear antigen) (a biomarker for proliferation) in these cell lines (Figure 1C). For p53, cyclin D1, LC3B, and FAS, the evoked changes tended to differ among these cell lines. Notably, the cleaved PARP fragment was significantly increased in RL95-2 cells, which is consistent with current MTT data.

**Figure 2:**
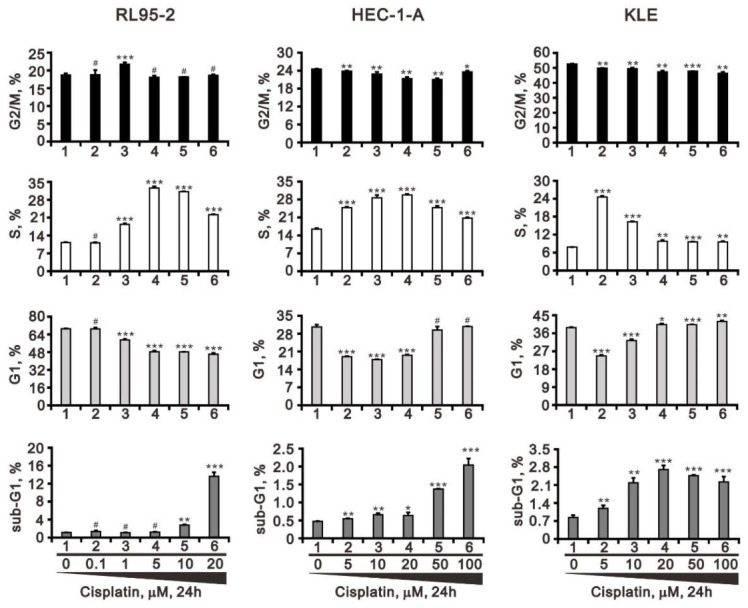
Effects of cisplatin on the cell cycle profile of human endometrial cancer cells. RL95-2, HEC-1-A, and KLE cells were incubated for 24 h with the indicated concentrations of cisplatin and then subjected to flow cytometric cell cycle profile analysis. Trend of three independent experiments was shown. ^#^ *p* > 0.05, * *p* < 0.05, ** *p* < 0.01, and *** *p* < 0.001 (Student’s *t*-tests).

Because the cell cycle related proteins p53 and cyclin D1 were differentially affected among the three cell lines, cell cycle profiles were determined to assess the changes of individual cell cycle phases (Figure 2). We found that the subG1 population was dose-dependently elevated in RL95-2 cells. In addition, the G1 population was dose-dependently suppressed while the S population was increased among RL95-2 cells. In HEC-1-A and KLE cells, we observed the minor and significantly increasing subG1 populations, the decreasing G1 populations in lower dosages and back to normal level in higher dosages, the increasing S populations, and the decreasing G2/M populations. Changes in the populations of cell cycle profile among HEC-1-A and KLE cells appeared unrelated to cisplatin dosage.

### 3.2. Perturbation of Reactive Oxygen Species (ROS) and Senescence by Cisplatin

**Figure 3:**
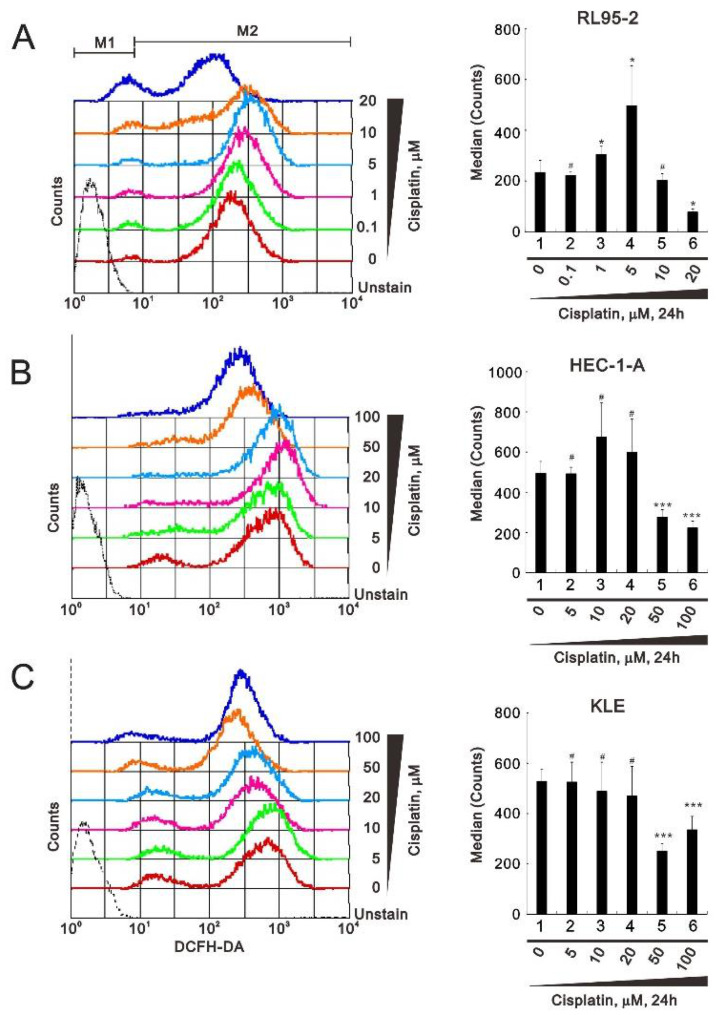
Effects of cisplatin on reactive oxygen species (ROS) levels in human endometrial cancer cells. (**A**–**C**) RL95-2 (**A**), HEC-1-A (**B**), and KLE (**C**) cells were incubated for 24 h with the indicated concentrations of cisplatin in the presence of 10 μM DCFH-DA (2′,7-dichlorofluorescein diacetate) and assayed using a flow cytometer. A representative and trend of three independent experiments were shown. ^#^ *p* > 0.05, * *p* < 0.05, ** *p* < 0.01, and *** *p* < 0.001 (Student’s *t*-tests).

Cisplatin induces ROS that trigger cell death [27]. We therefore compared the induction of ROS by cisplatin in RL95-2, HEC-1-A, and KLE cells. Using flow cytometry with DCFH-DA (2′,7-dichlorofluorescein diacetate), we found that at lower concentrations, cisplatin increased ROS generation, but at higher cisplatin concentrations ROS levels were suppressed to about 50% of control in RL95-2, HEC-1-A, and KLE cells (Figure 3). The maximal median ROS levels were elicited with 5 μM cisplatin in RL95-2 cells (Figure 3A), 10 μM cisplatin in HEC-1-A cells (Figure 3B), and 5 μM cisplatin in KLE cells (Figure 3C). The trend of ROS change was shown in each right panel, suggesting that higher (over 20 μM) cisplatin concentrations decreased ROS generation in all tested cells.

**Figure 4:**
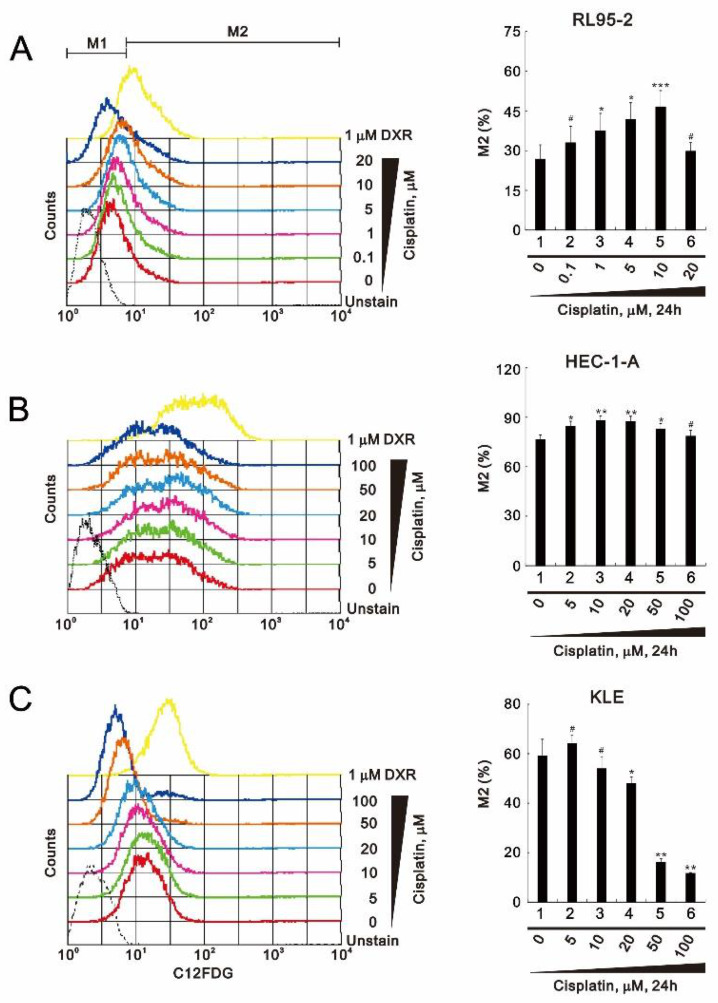
Effects of cisplatin on cellular senescence in human endometrial cancer cells. (**A**–**C**) RL95-2 (**A**), HEC-1-A (**B**), and KLE (**C**) cells were incubated for 24 h with the indicated concentrations of cisplatin in the presence of 33 μM C12FDG (5-dodecanoylaminofluorescein di-β-D-galactopyranoside) and assayed using a flow cytometer. A representative and trend of three independent experiments were shown. ^#^ *p* > 0.05, * *p* < 0.05, ** *p* < 0.01, and *** *p* < 0.001 (Student’s *t*-tests).

ROS play a key role in the induction of senescence in vitro and in vivo [28]. Flow cytometric analysis with C_12_FDG (5-dodecanoylaminofluorescein di-β-D-galactopyranoside) showed that cisplatin significantly increased cellular senescence at 10 μM cisplatin in RL95-2 cells, but it then declined to baseline at 20 μM (Figure 4A). In HEC-1-A cells, at concentrations up to 20 μM, cisplatin increased cellular senescence slightly, but it then declined to baseline at higher concentrations (Figure 4B). Notably, cisplatin dose-dependently decreased cellular senescence in KLE cells (Figure 4C). The trend of senescence change is shown in each panel on the right.

### 3.3. The Effects of Oxaliplatin on Specific Proteins and ROS in Endometrial Cancer Cells

**Figure 5:**
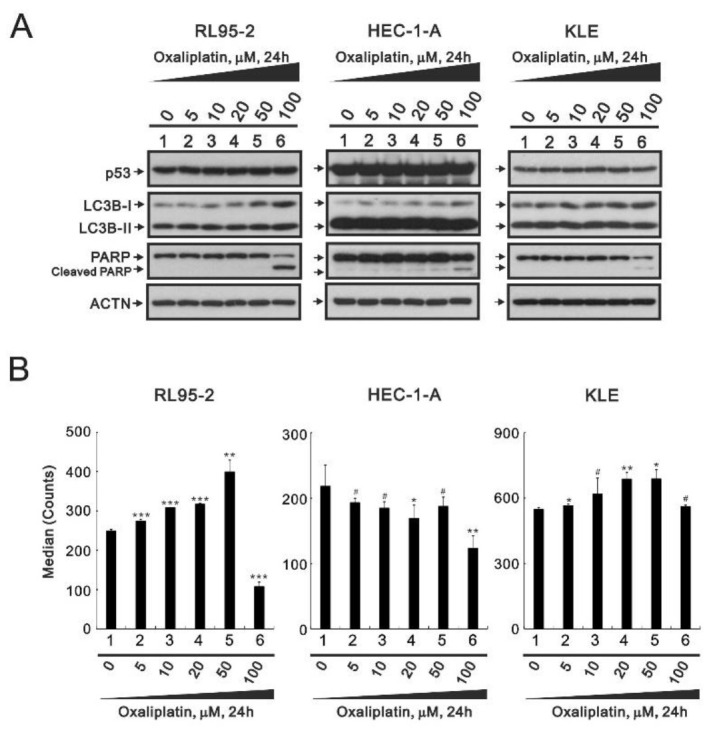
Responsiveness of human endometrial carcinoma cells to oxaliplatin. (**A**) RL95-2, HEC-1-A, and KLE cells were incubated for 24 h with the indicated concentrations of oxaliplatin, after which their cell lysates were subjected to western blot analysis using antibodies against the indicated proteins. ACTN was the protein loading control. (**B**) RL95-2, HEC-1-A, and KLE cells were incubated for 24 h with the indicated concentrations of oxaliplatin in the presence of 10 μM DCFH-DA and assayed using a flow cytometer. Trend of three independent experiments was shown. ^#^ *p* > 0.05, * *p* < 0.05, ** *p* < 0.01, and *** *p* < 0.001 (Student’s *t*-tests).

Based on the differential cytotoxic effect of cisplatin and oxaliplatin in RL95-2 cells (Figure 1B), we checked the potential cytotoxic effect of oxaliplatin in RL95-2, HEC-1-A, and KLE cells. Western blot analyses showed that oxaliplatin dose-dependently differentially increased expression of the cleaved PARP fragment in RL95-2, HEC-1-A, and KLE cells (Figure 5A). In addition, changes of the ratio of II/I of LC3B suggest oxaliplatin suppresses autophagy in RL95-2 cells.

Measurement of DCFH-DA signals showed that oxaliplatin initially induced dose-dependent increases in ROS levels, but with further increases in the oxaliplatin concentration, ROS levels declined to baseline levels or even lower in RL95-2 cells (Figure 5B). We observed a downward trend in HEC-1-A cells and a trend which increased before 50 μM and descended to the basal level at 100 μM in KLE cells.

### 3.4. Characterization of the RL95-2 and HEC-1-A Cell Types

**Figure 6:**
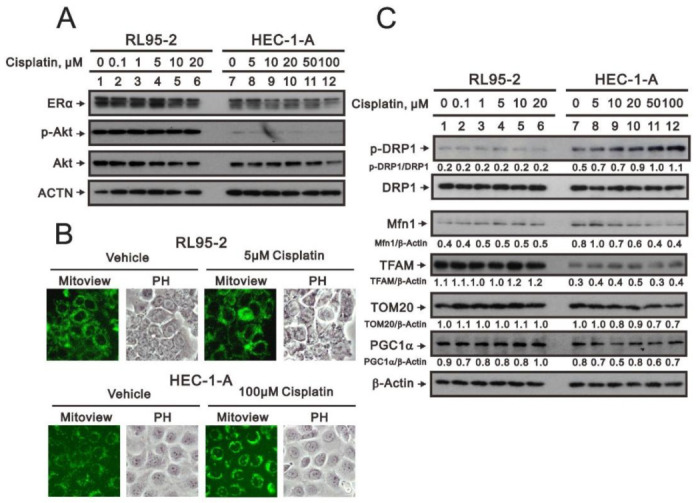
The characteristics of RL95-2 and HEC-1-A endometrial carcinoma cells. RL95-2 and HEC-1-A cells were incubated for 24 h with the indicated concentrations of cisplatin. (**A**,**C**) Cell lysates were subjected to western blot analysis using antibodies against the indicated proteins. Actin (ACTN) (**A**) or β-actin (**C**) were the protein loading controls. (**B**) RL95-2 and HEC-1-A cells were respectively incubated with 5 μM and 100 μM cisplatin for 24 h, after which the cells were washed, incubated with 10 nM MitoView^TM^ Green and observed under a LeadView 2800AC-FL microscope equipped with a 400× objective.

Given the differential responsiveness of RL95-2 (type I) and HEC-1-A (type II) cells to cisplatin, we reconfirmed which tumor type these two cell lines were derived from [29]. We first used Western blotting to assess levels of estrogen receptor alpha (ERα and the status of PTEN (based on levels of p-Akt). Type II endometrial cancer cells express wild-type PTEN, which negatively regulates Akt phosphorylation. In RL95-2 cells, we observed higher levels of ERα and p-Akt than in HEC-1-A cells (Figure 6A), supporting that RL95-2 are type I while HEC-1-A are type II endometrial cancer cells.

The frequent recurrence of endometrial cancers and the drug resistance of the recurrent tumors remain obstacles to treatment. Moreover, mitochondrial dysfunction has been implicated in the resistance of these tumors to treatment [30,31,32,33]. The involvement of mitochondria in cisplatin resistance has been shown to involve mitochondrial dynamics and bioenergetics metabolism. Here, we used MitoView dye to evaluate the fusion-fission status of mitochondria in RL95-2 and HEC-1-A cells. In the presence of an effective cytotoxic dosage of cisplatin (5 μM for RL95-2 and 100 μM for HEC-1-A cells), the fission phenotype was observed in HEC-1-A cells but not RL95-2 cells (Figure 6B). Two outer mitochondrial membrane proteins, dynamin-related protein 1 (DRP1) and a membrane-anchored dynamin family member, MFN1, are involved in the processes of mitochondrial fission and fusion [34,35]. Our Western blotting data showed that cisplatin increased the p-DRP1/DRP1 ratio and decreased levels of MFN1 in HEC-1-A cells (Figure 6C), but induced no apparent changes in these two proteins in RL95-2 cells. Our current data suggest that cisplatin might have the ability to switch HEC-1-A cells to the fission status.

Peroxisome-proliferator-activated receptor γ co-activator-1α (PGC-1α) and mitochondrial transcription factor A (TFAM) are two key mitochondrial respiratory and biogenic factors for mitochondrial respiratory function [36,37]. Figure 6C also shows that cisplatin tended to increase levels of both PGC-1α and TFAM in RL95-2 cells and to reduce levels of TOM20, another key outer mitochondrial membrane protein, in HEC-1 -A cells. Hence, our current findings suggest that bioenergetic metabolism of mitochondria might be enhanced in cisplatin-treated RL95-2 cells.

### 3.5. Screening Cisplatin in Combination with Repurposed dRugs in RL95-2 and HEC-1-A Cells

**Figure 7:**
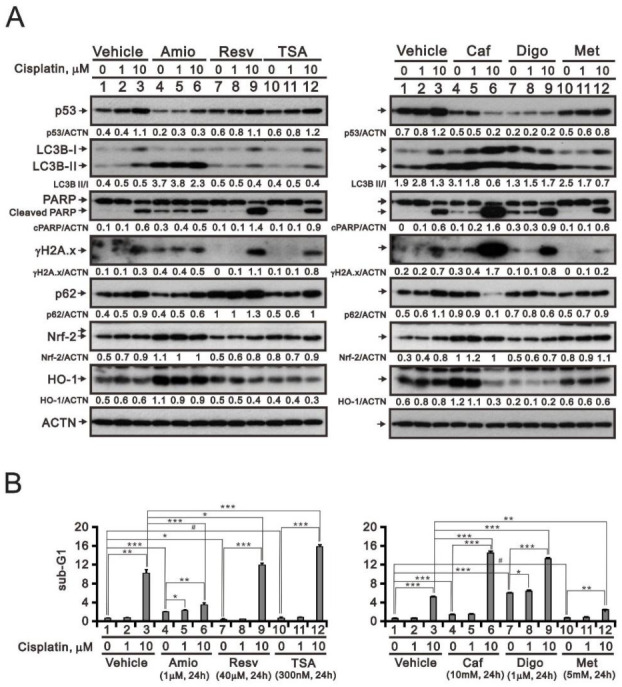
Responsiveness in RL95-2 human endometrial carcinoma cells to cisplatin in combination with selected repurposed drugs. (**A**,**B**) RL95-2 cells were incubated for 24 h with the indicated concentrations of cisplatin plus1 μM amiodarone (Amio), 40 μM resveratrol (Resv), 300 nM trichostatin A (TSA), 10 mM caffeine (Caf), 1 μM digoxin (Digo), or 5 mM metformin (Met). (**A**) Cell lysates were subjected to Western blot analysis using antibodies against the indicated proteins. ACTN was the protein loading control. (**B**) Flow cytometric cell cycle profile analysis of the effect of the indicated treatments on the SubG1 population. Trend of three independent experiments was shown. ^#^ *p* > 0.05, * *p* < 0.05, ** *p* < 0.01, and *** *p* < 0.001 (Student’s *t*-tests).

**Figure 8:**
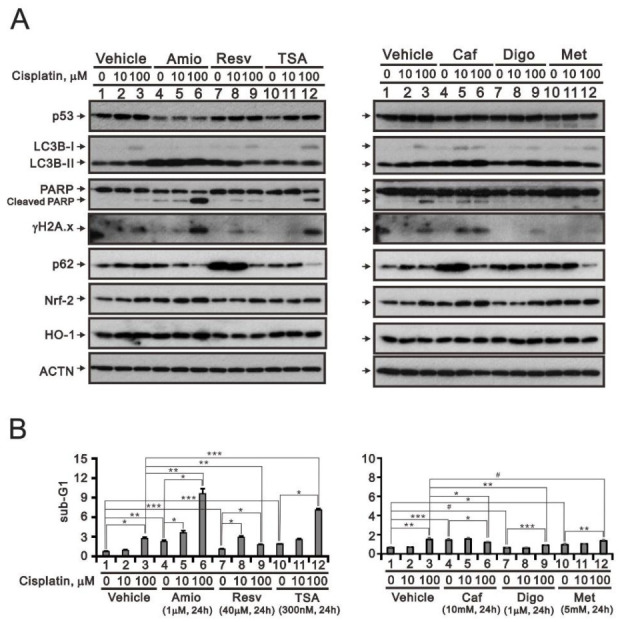
Responsiveness in HEC-1-A human endometrial carcinoma cells to cisplatin in combination with selected repurposed drugs. (**A**,**B**) HEC-1-A cells were incubated for 24 h with the indicated concentrations of cisplatin plus 1 μM amiodarone (Amio), 40 μM resveratrol (Resv), 300 nM trichostatin A (TSA), 10 mM caffeine (Caf), 1 μM digoxin (Digo), or 5 mM metformin (Met). (**A**) Cell lysates were subjected to western blot analysis using antibodies against the indicated proteins. ACTN was the protein loading control. (**B**) Flow cytometric cell cycle profile analysis of the effect of the indicated treatments on the SubG1 population. Trend of three independent experiments was shown. ^#^ *p* > 0.05, * *p* < 0.05, ** *p* < 0.01, and *** *p* < 0.001 (Student’s *t*-tests).

The challenge to management of type II or recurrent endometrial cancer is the lack of an efficacious chemotherapy regimen [38]. The aim of drug repurposing is the primary to overcome resistance to cisplatin while reducing the drug’s toxicity in cancer therapy. In RL95-2 cells, the entire valine codon 218 of p53 is deleted, while HEC-1-A and KLE cells respectively exhibit R248N and R175H point mutations [39]. These changes are located in the DNA binding domain of p53 and apparently affect its transcriptional activity in endometrial cancer cells. Although p53 plays a vital role in the response of cisplatin-induced DNA damage, p53-negative cells also respond to cisplatin-induced DNA damage, suggesting the existence of an alternative pathway to cell death. However, we focused on the potential antitumor functions of several repurposed drugs on the basis that p53 alterations and DNA damage are involved in the mediation of endometrial cancer pathogenesis. The drugs tested for repurposing in this study were chosen according to our previous work [19,20,21,22,23]. We chose cisplatin-treated RL95-2 and HEC-1-A cells to test the efficacy of cisplatin in combination with amiodarone, resveratrol, TSA, caffeine, digoxin, or metformin (Figure 7 and Figure 8). Western blot analysis showed that by increasing levels of cleaved PARP fragment and the cell population in subG1 phase of the cell cycle, resveratrol, TSA, caffeine, and digoxin may increase the apoptotic process of cisplatin toward RL95-2 cells (Figure 7), while amiodarone and TSA may increase the cisplatin apoptotic process toward HEC-1-A cells (Figure 8). Evoked increases in the levels of the DNA damage marker γH2A.x were consistent with the higher levels of cleaved PARP fragments and greater subG1 phase populations in both cell lines. Levels of p53; Nrf2 and HO-1, two anti-oxidative proteins; and LC3B-p62, a protein involved in autophagy; were not consistent with the higher levels of cleaved PARP fragments or the larger populations of subG1 phase cells in the two cell lines under current experimental conditions. 

**Figure 9:**
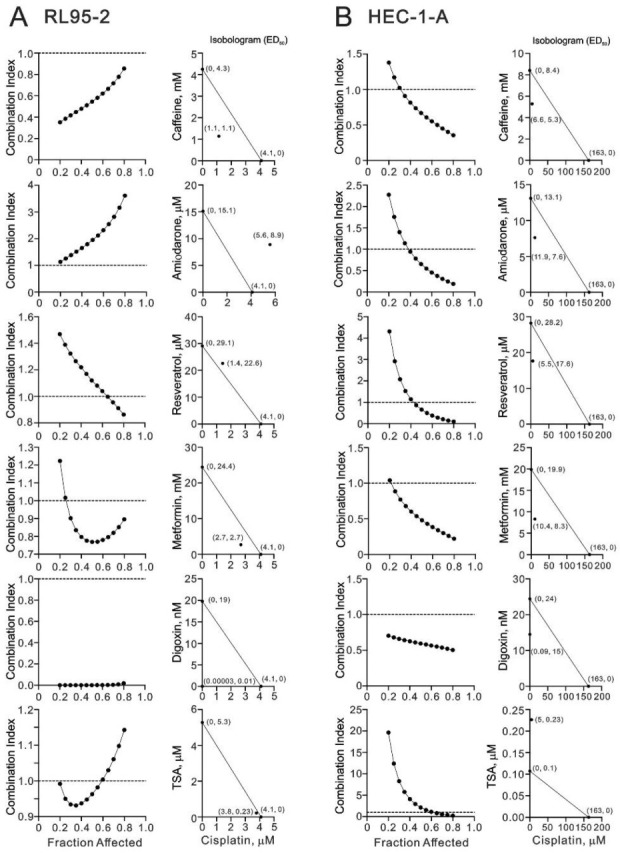
The combination index of cisplatin with caffeine, amiodarone, resveratrol, metformin, digoxin, or TSA in RL95-2 and HEC-1-A cells. (**A**) RL95-2 and (**B**) HEC-1-A cells were treated with caffeine dose: 0, 0.156, 0.313, 0.625, 1.25, 2.5, 5, 10, 20, 40 mM, amiodarone dose: 0, 0.125, 0.25, 0.5, 1, 2, 4, 8, 16, 32 μM, resveratrol dose: 0, 1.25, 2.5, 5, 10, 20, 40, 80, 160, 320 μM, metformin dose: 0, 0.15625, 0.3125, 0.625, 1.25, 2.5, 5, 10, 20, 40 mM, digoxin dose: 0, 0.03125, 0.0625, 0.125, 0.25, 0.5, 1, 2, 4, 8 μM, or TSA dose: 0, 9.375, 18.75, 37.5, 75, 150, 300, 600, 1200, 2400 nM, combined with cisplatin dose: 0, 0.625, 1.25, 2.5, 5, 10, 20, 40 μM for RL95-2 cells and 0, 1.563, 3.125, 6.25, 12.5, 25, 50, 100 μM for HEC-1-A cells. Metabolic activity was measured by the MTT method. The combination index of cisplatin plus specific drug in (**A**) RL95-2 and (**B**) HEC-1-A cells. Isobolograms (ED_50_) of cisplatin was calculated using CalcuSyn software.

Based on these half maximal inhibitory concentrations (IC_50_) values and the classic experimental design [25], we designed these combinations of concentrations and calculated the combination index (CI) between cisplatin and caffeine, amiodarone, resveratrol, metformin, digoxin, or TSA in RL95-2 and HEC-1-A cells. CI < 1, as a synergistic effect, was observed in most of testing combination of cisplatin plus drugs in RL95-2 and HEC-1-A cells (Figure 9), except that the combination of cisplatin plus amiodarone was CI > 1 in RL95-2 cells. We further analyzed the ED_50_ (median effective dose) of two combinations from the data calculated for the CI. The therapeutic concentrations of 4.1 μM cisplatin were decreased to 1.1 μM (at 1.1 mM caffeine), 1.4 μM (at 22.6 μM resveratrol), 2.7 μM (at 2.7 mM metformin), 0.00003 μM (at 0.01 nM digoxin), and 3.8 μM (at 0.23 μM TSA) in RL95-2 cells (Figure 9A) and 163 μM cisplatin were decreased to 6.6 μM (at 5.3 mM caffeine), 11.9 μM (at 7.6 μM amiodarone), 5.5 μM (at 17.6 μM resveratrol), 10.4 μM (at 8.3 mM metformin), 0.09 μM (at 15 nM digoxin), and 5 μM (at 0.23 μM TSA) in HEC-1-A cells (Figure 9B). The therapeutic concentration of 4.1 μM cisplatin was increased to 5.6 μM (at 8.9 μM amiodarone), suggesting the combination was antagonistic effect (Figure 9A). These findings suggest that the benefit of the clinical application of cisplatin plus synergistic drugs might be one of the potential choices to overcome the current resistance to cisplatin in type II or recurrent endometrial cancer.

**Figure 10:**
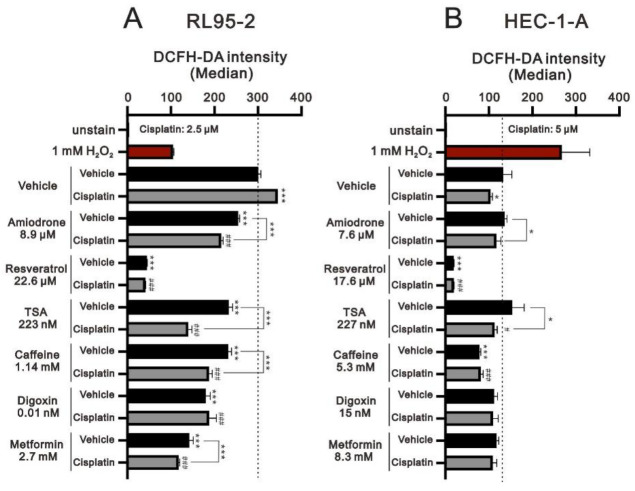
Effects of the combination of cisplatin plus repurposing drugs on ROS levels in human endometrial cancer cells. (**A**) RL95-2 and (**B**) HEC-1-A cells were incubated for 1.5 h with the indicated concentrations of cisplatin, repurposing drug, and cisplatin plus indicated repurposing drug in the presence of 10 μM DCFH-DA and assayed using a flow cytometer. Trends of three independent experiments were shown. * *p* < 0.05 and *** *p* < 0.001 for vehicle or repurposing drug; ^#^ *p* < 0.05 and ^###^ *p* < 0.001 for cisplatin (Student’s *t*-tests).

We further examined the level of ROS with the indicated concentration of cisplatin plus repurposing drug via our CI data from Figure 9 in RL95-2 and HEC-1-A cells (Figure 10). We applied 2.5 μM cisplatin in RL95-2 cells (Figure 10A) and 5 μM cisplatin in HEC-1-A cells (Figure 10B) with indicated concentration of amiodarone, resveratrol, TSA, caffeine, digoxin, and metformin. Compared positive control hydrogen peroxide with vehicle, our data showed that the basal ROS level was much higher and less responsive to hydrogen peroxide in RL95-2 cells and the basal ROS level was much lower and more responsive to hydrogen peroxide in HEC-1-A cells. Consistently, cisplatin elevated the basal ROS level in RL95-2 cells and decreased the basal ROS level in HEC-1-A cells. Amiodarone, resveratrol, TSA, caffeine, digoxin, and metformin significantly decreased the basal and cisplatin-induced ROS level in RL95-2 cells (Figure 10A). In HEC-1-A cells, TSA elevated the basal ROS level and resveratrol, caffeine, digoxin, and metformin decreased the basal ROS level. The ROS suppressed by cisplatin were reversed by the combination of amiodarone, TSA, digoxin, and metformin and was further significantly suppressed by the combination of resveratrol and caffeine (Figure 10B). 

## 4. Discussion

Most type I low-grade endometrial endometrioid carcinomas are detected early and have a favorable prognosis; however, high-grade endometrial endometrioid carcinomas have much poorer prognosis. Only about 10% of endometrial cancers are type II, but they typically present at an advanced stage, account for approximately 50% of recurrences, and have a poor prognosis [5]. Patients of endometrial cancer are typically elderly with multiple comorbidities, which makes aggressive cytotoxic therapy potentially hazardous. Consequently, there is a pressing need to define optimal treatment strategies for advanced and recurrent disease and to personalize therapy based on individual tumor and patient characteristics. Here, we found that RL95-2 (type I) cells are more sensitive to cisplatin than HEC-1-A (type II) or KLE (type II) cells. The cytotoxicity of cisplatin in RL95-2 cells may be the result of induced perturbations in the cell cycle, ROS production and autophagy, as well as induction of cellular senescence and DNA damage. Similar alterations in the cell cycle, ROS, autophagy, and senescence were also observed in HEC-1-A and KLE cells. In addition, suppression of p53 and/or cyclin D1 by cisplatin may also impact the responsiveness of HEC-1-A and KLE cells to cisplatin. Our screening of repurposed drugs for use in combination with cisplatin revealed that resveratrol, TSA, caffeine, and digoxin may increase the apoptotic process of cisplatin toward RL95-2 cells, while amiodarone and TSA may increase its apoptotic process toward HEC-1-A cells. HEC-1-A cells were sensitive to the hydrogen peroxide treatment, suggesting that endogenous anti-oxidative system fails to response this oxidative stress. In RL95-2 cells, higher endogenous ROS level might have a relative efficient anti-oxidative system to ameliorate the stress of exogenous hydrogen peroxide. Many studies demonstrated that cancer cells were sensitive to cisplatin not to oxaliplatin since DNA adducts formed by oxaliplatin are not recognized by the DNA mismatch repair system, such as hMSH2 or hMLH1 [40].

Previous studies have provided a two-step explanation for how oxidative stress may lead to endometrial carcinoma [12]. In the first step, oxidative stress-induced DNA damage enhances cell apoptosis among endometrial cells and production of antioxidants for survival. In the second step, cancer pathogenesis is associated with persistent antioxidant production favoring a pro-tumoral microenvironment [12]. Thus, upregulation of antioxidant function in endometriosis may enhance cell survival and facilitate subsequent malignant transformation [41]. Increasing ROS by pharmacological means could potentially sensitize endometrial cancer cells to cisplatin and overcome resistance. Many United States Food and Drug Administration-approved anti-cancer drugs efficiently eliminate cancer cells and drug resistance by increasing ROS production [42,43]. Here, cisplatin differentially increased ROS in RL95-2, HEC-1-A, and KLE cells. Excessive ROS may have induced apoptosis via cleavage of PARP in all three cell types studied.

Once oxidative stress causes DNA damage, cell cycle checkpoints are activated. Our data showed that cisplatin suppressed the G1 phase population and induced the S phase population in RL95-2 cells, but not HEC-1-A or KLE cells. This suggests delay of cell cycle progression led to RL95-2 cell death. However, cells have developed a number of antioxidant systems, including superoxide dismutase, catalase and glutathione peroxidase, to limit ROS production, inactivate ROS, and repair cell damage. Further study will be needed to determine whether oxidative stress occurs when the balance between ROS production and antioxidant defense is disrupted under conditions such as those in the present study.

Endometrial cancer is a heterogeneous disease with several subtypes that differ on prognosis and molecular background [44]. The response to chemotherapy (e.g., cisplatin) and whether or not there is drug resistance are the most critical factors influencing the prognosis and overall survival of endometrial cancer patients. The challenge to the management of recurrent endometrial cancer is the lack of an efficacious chemotherapeutic drug regimen. Our data indicate that RL95-2 cells were more sensitive to cisplatin than HEC-1-A or KLE cells and the longer cisplatin treatment of KLE cells were more sensitive to HEC-1-A cells (supplementary Figure S2). This suggests that a comparison between RL95-2 and HEC-1-A may shed light on the mechanism of cisplatin resistance. In addition to the generation of ROS, changes in mitochondrial dynamics and bioenergetics metabolism have been shown to be involved in mediating resistance to cisplatin [30,32]. Our findings suggest that the different responses of RL95-2 and HEC-1-A cells to cisplatin reflect, in part, differences in the drug’s effects on mitochondrial dynamics and bioenergetics. This highlights the need to base decisions about a patient’s cisplatin resistance status on the genetics of their particular endometrial cancer. A major challenge to understanding cisplatin resistance is that a wide array of individual resistance mechanisms has been identified (in PubMed, more than 15,565 papers match the search term “cisplatin resistance”).

The drugs tested for repurposing in the present study were chosen based on our previous work [19,20,21,22,23]. p53 is a short-lived protein that is activated (phosphorylated) by ataxia telangiectasia mutated on the DNA damage signal [45]. The activated p53, in turn, activates Mdm2, which is an E3 ubiquitin ligase [46]. p53 can also transactivate genes involved in cell-cycle progression, DNA repair and apoptosis [47]. Mutant p53 proteins mostly lose their tumor-suppressive functions and may exert dominant-negative activities, but may also gain new oncogenic properties [48]. Despite the high cure rate, some germ cell tumors do develop resistance, and this seems to be due primarily to high levels of cytoplasmic p21 [49]. In general, p21 is a downstream mediator of cell death, but cytosolic localization of p21 protects cells from Fas-mediated apoptosis [50]. A well-known target of p53 is p21, suggesting the differential responsiveness of RL95-2, HEC-1-A, and KLE cells to cisplatin may reflect differences in the subcellular localization of p53 and p21in these cells.

It is sometimes difficult to classify endometrial carcinoma subtypes as type I or type II. Histological classification is currently the gold standard for patient stratification. However, molecular studies have yielded promising results, which may provide important information for improving prognostic accuracy and predicting responses to novel therapies. Recently, patient-derived xenograft models have been shown to reliably preserve the genetic, histological, and phenotypical characteristics of the primary tumor with a 60–80% graft success rate [51,52]. However, the primary culture of endometrial carcinoma cells from patients may be a more direct way to determine the responsiveness to cisplatin alone and in combination with other agents. Based on our current findings, we would expect that patients with tumors like the RL95-2 type with higher endogenous ROS level would be sensitive to cisplatin monotherapy or, if necessary, combination therapy with caffeine, metformin, TSA, or digoxin. On the other hand, those with tumors similar to the HEC-1-A type would be sensitive to cisplatin in combination with caffeine, amiodarone, resveratrol, metformin, digoxin, or TSA. In addition to endometrial cancers, other germ cell tumors, ovarian and testicular, have demonstrated that the histone deacetylase inhibitor family, including TSA, has been achieving good results in the treatment of germ cell tumors with cisplatin resistance via epigenetic mechanism [53,54]. However, our combination index experiments provide novel insights into caffeine, amiodarone, resveratrol, metformin, digoxin, or TSA to dramatically decrease the dosage of cisplatin in current therapeutic treatment. The challenge of side effects of amiodarone, metformin, and digoxin could be adjusted and improved for further clinical application. As evidence from clinical trials accumulates, we anticipate that beneficial repurposed drugs will become clinically available in the future.

## 5. Conclusions

We found that RL95-2 (type I) cells are more sensitive to cisplatin than HEC-1-A (type II) or KLE (type II) cells. The cytotoxicity of cisplatin in RL95-2 cells may be the result of induced perturbations in the cell cycle, ROS production and autophagy, as well as induction of cellular senescence and DNA damage. In addition, suppression of p53 and/or cyclin D1 by cisplatin may also impact the responsiveness of HEC-1-A and KLE cells to cisplatin. Our screening of repurposed drugs for the apoptotic process in combination with cisplatin revealed that TSA might be the only repurposed drug effective against our tested endometrial cancer cell types. Furthermore, the combination index suggests that caffeine, amiodarone, resveratrol, metformin, digoxin, and TSA potentially serve as cisplatin sensitizers. As evidence from clinical trials accumulates, we anticipate that beneficial repurposed drugs will become clinically available in the future.

## Data Availability

Not applicable.

## Supplementary Materials

The following are available online at https://www.mdpi.com/2075-1729/11/2/160/s1, Figure S1: Responsiveness of human endometrial carcinoma cells to oxaliplatin. HEC-1-A and KLE cells were incubated for 24 h with the indicated concentrations of oxaliplatin. Metabolic activity measured using the MTT method for oxaliplatin. Trend of three independent experiments was shown, Figure S2: Responsiveness of human endometrial carcinoma cells to cisplatin. HEC-1-A and KLE cells were incubated for 48 h with the indicated concentrations of cisplatin. Metabolic activity measured using the MTT method for cisplatin. Trend of three independent experiments was shown.

## Abbreviations

ACTN	α-actinin
C_12_FDG	5-dodecanoylaminofluorescein di-β-d-galactopyranoside
CI	combination index
DCFH-DA	2′,7-dichlorofluorescein diacetate
DMEM	Dulbecco’s modified Eagle’s medium
DRP1	dynamin-related protein 1
ED_50_	median effective dose
ER	Estrogen receptor
FACS	fluorescence- activated cell sorting
FBS	fetal bovine serum
FITC	fluorescein isothiocyanate
HO-1	heme oxygenase 1
IC_50_	half maximal inhibitory concentration
MFN1	membrane-anchored dynamin family member
MTT	thiazolyl blue tetrazlium bromide
PBS	phosphate buffered saline
PI	propidium iodide
PARP	poly-ADP-ribose polymerase
PGC-1α	Peroxisome-proliferator-activated receptor γ co-activator-1α
PI3K/AKT	Phosphatidylinosito 3-kinase/a serine/threonine protein kinase
PTEN	Phosphatase and Tensin Homology
ROS	reactive oxygen species
RPMI	Roswell Park Memorial Institute
TSA	trichostatin A
TFAM	mitochondrial transcription factor A
