# Supplementary material for: Mechanisms of Cisplatin in Combination with Repurposed Drugs against Human Endometrial Carcinoma Cells
Source: Life (Basel). 2021 Feb 19;11(2):160. doi: 10.3390/life11020160 (PMC7922822; doi:10.3390/life11020160)
Supplement: Supplementary file 1 [file life-11-00160-s001.pdf]

Supplementary data

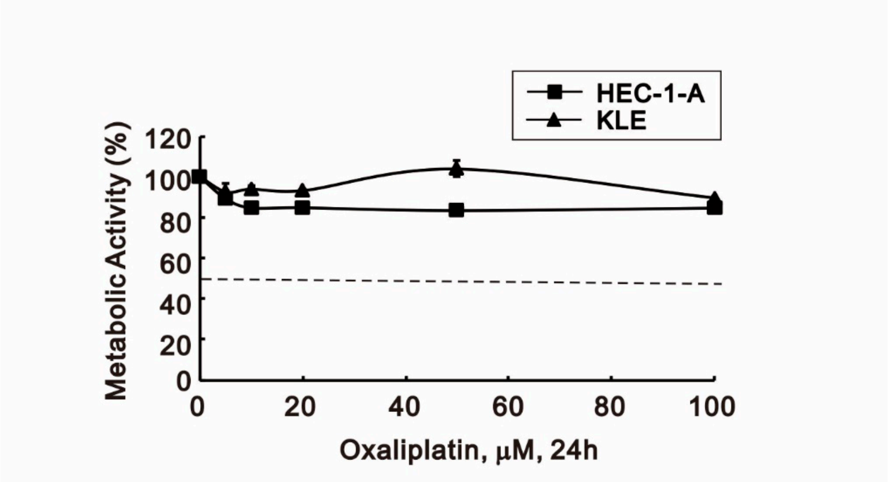

Figure S1. Responsiveness of human endometrial carcinoma cells to oxaliplatin. HEC-1-A and KLE cells were incubated for 24 h with the indicated concentrations of oxaliplatin. Metabolic activity measured using the MTT method for oxaliplatin. Trend of three independent experiments was shown.

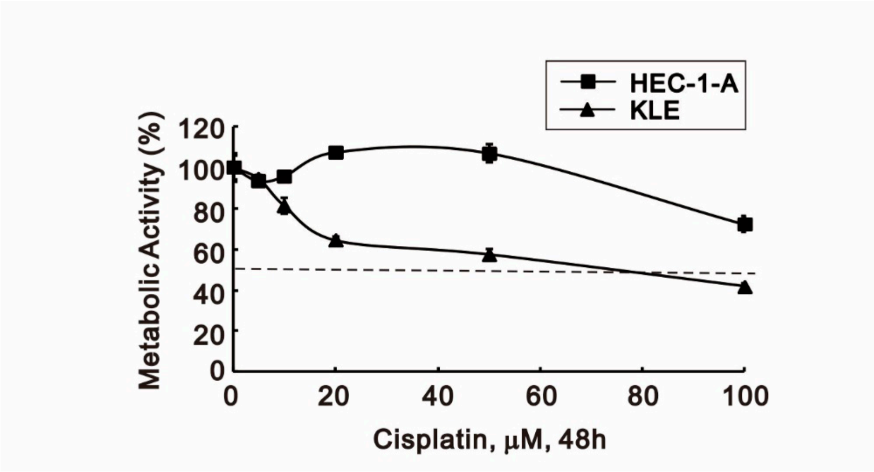

Figure S2. Responsiveness of human endometrial carcinoma cells to cisplatin. HEC-1-A and KLE cells were incubated for 48 h with the indicated concentrations of cisplatin. Metabolic activity measured using the MTT method for cisplatin. Trend of three independent experiments was shown.
